# NUT Carcinoma of the Lung：A Case report and Literature Analysis

**DOI:** 10.3389/fonc.2022.890338

**Published:** 2022-07-12

**Authors:** Rongshuang Zhao, Ze Hua, Xiaodong Hu, Qi Zhang, Jin Zhang, Jian Wang

**Affiliations:** ^1^ Department of Oncology, The Second Affiliated Hospital of Zhengzhou University, Zhengzhou, China; ^2^ Department of Medical Imaging, Zhengzhou University People’s Hospital (Henan Provincial People’s Hospital), Zhengzhou, China; ^3^ Department of Precision Medicine Center, The Zhengzhou University, Zhengzhou, China; ^4^ Department of Thoracic Surgery, The Second Affiliated Hospital of Zhengzhou University, Zhengzhou, China

**Keywords:** nuclear protein in testis, midline carcinoma, lung, case report, literature analysis

## Abstract

NUT carcinoma is a rare, highly aggressive cancer that feature as the rearrangement of the nuclear protein in the testis (NUT) gene on chromosome 15q14, and its pathogenesis and treatment is not yet clear. In this case, we report a 40-year-old male patient who was diagnosed with primary pulmonary NUT carcinoma in The Second Affiliated Hospital of Zhengzhou University. A tumor was found at the right hilus pulmonis when his physical examination with chest pain for half a month. Histopathology confirmed by fluorescence *in situ* hybridization technique for the NUT carcinoma. After chemotherapy, radiotherapy, immunotherapy, and targeted therapy were given, the patient died. The overall survival time was 4.7 months. Combined with the existing literature, we retrospective report the clinical and pathological characteristics and treatment strategies of the rare lung NUT carcinoma.

## Introduction

NUT carcinoma (nuclear protein in testis carcinoma) is a poorly differentiated carcinoma accompanied by a rearrangement of the NUT (nuclear protein in testis) gene on chromosome 15. The disease was initially considered to be a unique tumor in children and adolescents.However, recent studies have found that NUT cancer can occur at any age (1, 2). The disease is very rare. By consulting domestic and foreign literature, we found that there are no more than 100 clinically reported cases of the disease (3–7). The tumor is more common in the midline, and the common chromosomal translocation is t(15;19). Therefore, this cancer is also called midline cancer (NUT middle carcinoma, NMC), or t(15;19) carcinoma (8). At present, the pathological features, imaging findings and standard treatment regimens of NUT cancers that originate in the lung are still unclear. This article reports on the diagnosis and treatment process of a NUT cancer patient, andreviews the literature in order to provide a reference for clinical diagnosis and treatment.

## Case Presentation

**Figure 1:**
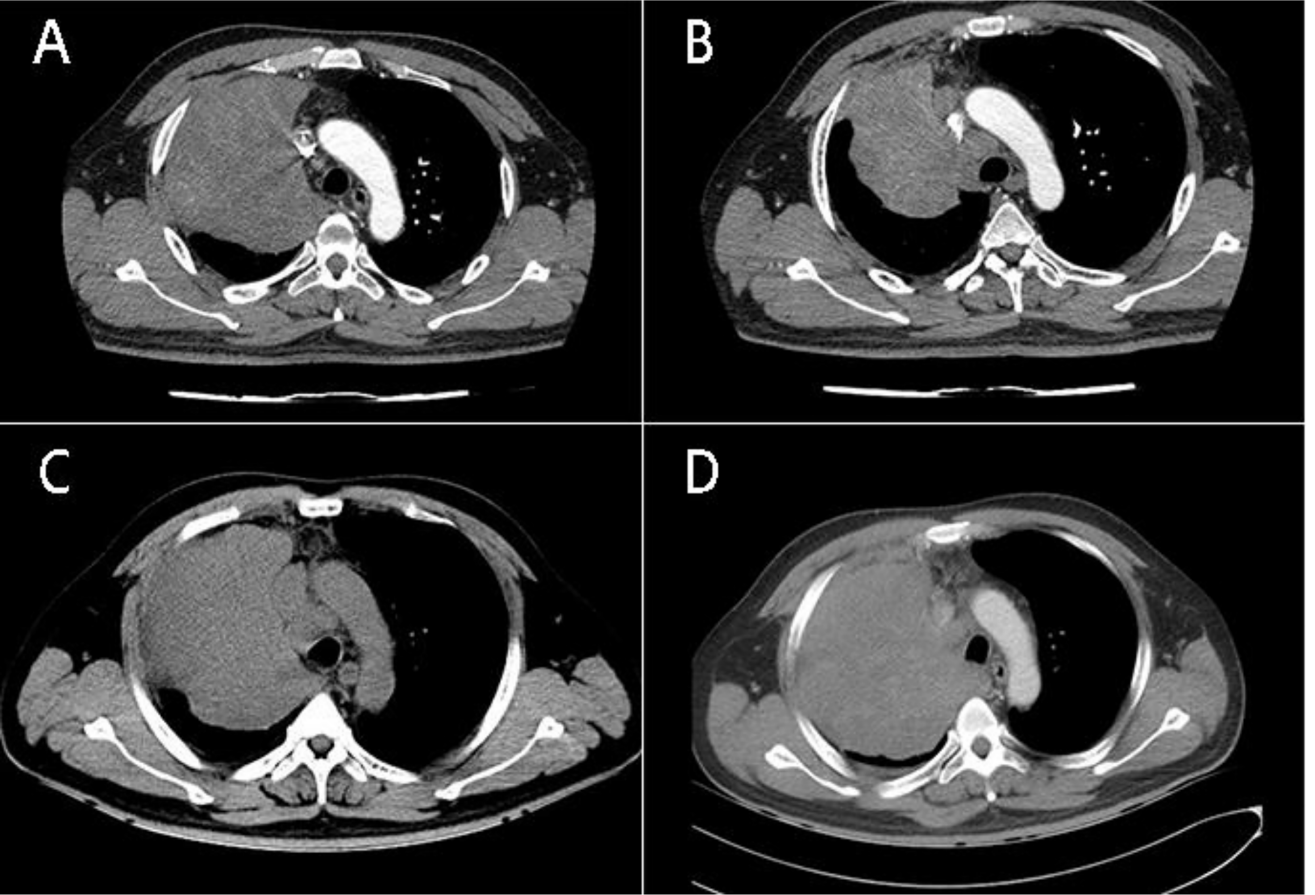
**(A)** CT of the patient’s chest (July 10, 2018) shows: Massive soft tissue-like density shadows can be seen near the hilar of the right upper lung, the size is about 11.4cm* 10.7cm, the enhanced scan is uneven and moderately enhanced; **(B)** The patient’s chest CT (September 11, 2018) after VAI regimen chemotherapy showed that the size of the right lung mass was about 8.9*8.1 cm, which was smaller than before, but the mediastinal lymph nodes were enlarged; **(C)** (September 30,2018) The size of the right lung mass is about 12.1cm*10.5 cm, which is larger than before. **(D)** (October 11, 2018) The soft tissue-like density shadow of the right upper lobe of the right lung mass, the right hilum is compressed, the main trachea of the right lower lung is compressed and narrowed, and the righthilar and mediastinum have multiple enlarged lymph nodes.

**Figure 2:**
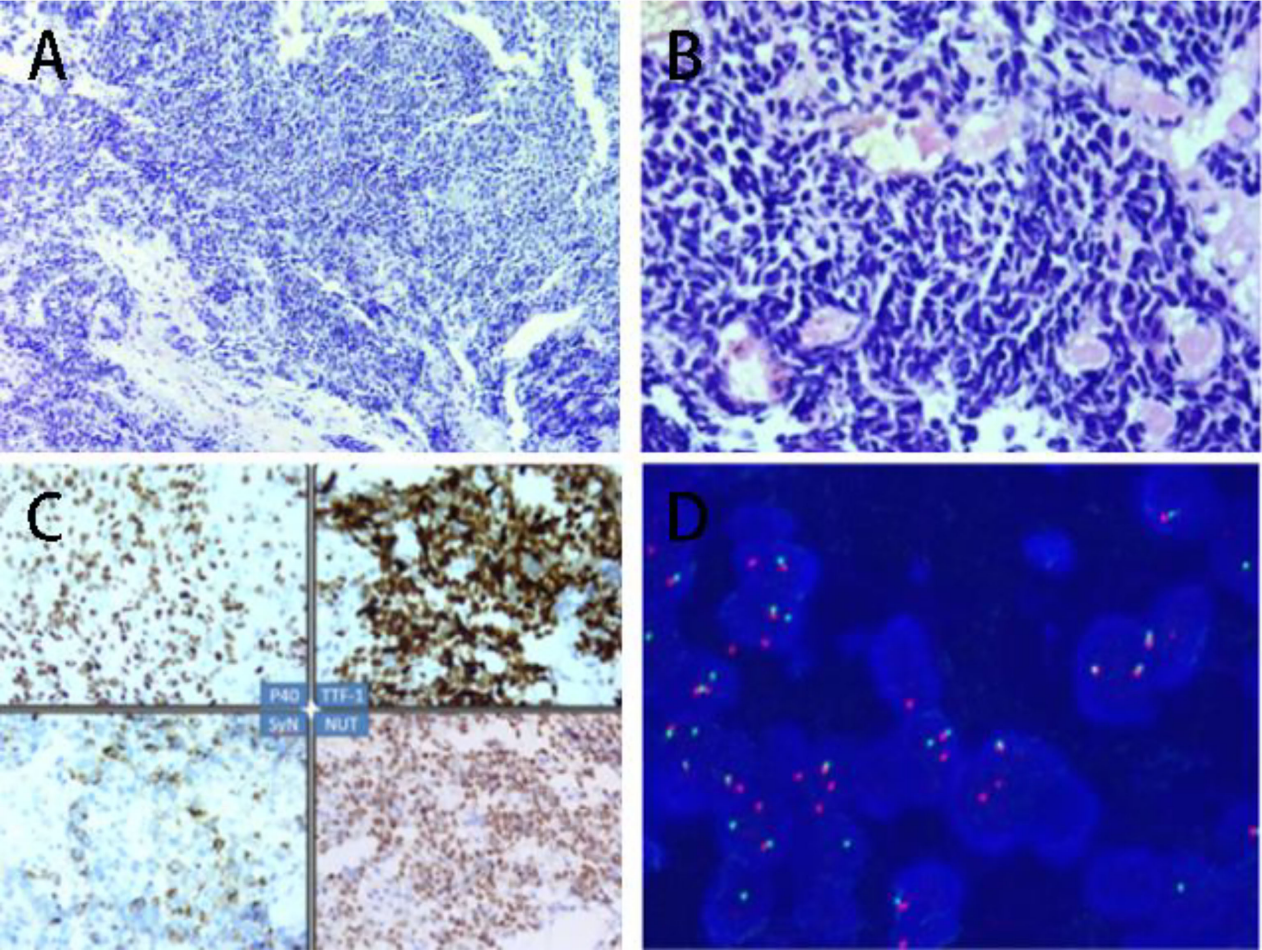
**(A)** Tumor small-medium size, fine nuclear staining, no nucleoli (HE, 10X); **(B)** Tumor small-medium size, fine nuclear staining, no nucleoli (HE, 40X); **(C)** 1.P40 positive(Immunohistochemical staining); 2. TTF-1 positive (Immunohistochemical staining); 3. SyN positive (Immunohistochemical staining); NUT positive (Immunohistochemical staining); **(D)** Fluorescence *in situ* hybridization (FISH) display: 45% of tumor cells show red and green separation signals (DAPI color development, 1000X).

A 40-year-old male patient presented with chest pain for half a month. At the time of presentation, the patient had no accompanying symptoms such as cough, sputum, hemoptysis, etc. The patient has no previous history of alcohol and tobacco addiction and no special family history. Computer tomography (computed tomography, CT) showed that there was a mass of soft tissue-like density shadow in the right upper lung near the hilar, about 11.4cm*10.7cm. The boundary between the lesion and the superior vena cava and right pulmonary artery is not clear. Enhanced scanning lesions were uneven and moderately enhanced, and multiple swollen lymph nodes in the mediastinum (**Figure 1A**). Bronchoscopic biopsy was performed at the local hospital, and small biopsy tissues with a diameter of 0.4 cm were sent for examination. The pathological diagnosis was: (right lung) small cell carcinoma. The result of the pathological consultation at the Second Affiliated Hospital of Zhengzhou University: poorly differentiated cancer, tumor cells are undifferentiated, small-medium size cells, round, oval, or short spindle-shaped nuclei, fine nuclear chromatin, no obvious nucleoli (**Figures 2A, B**). According to the morphology, the first consideration is small cell carcinoma. However, the immunohistochemically labeled tumor cells express squamous cell carcinoma markers such as P40 and CK5/6, and also the adenocarcinoma marker TTF-1. NUT has suspicious positive cells (**Figure 2C**) simultaneously. Thus, lung NUT Cancer cannot be excluded. Therefore, further fluorescence *in situ* hybridization (FISH) detection is needed to confirm the diagnosis. Immunohistochemistry results: epithelial markers AE1/AE3 (membrane +), CK7 (-), CK5/6 (about 30% tumor cells scattered +); neuroendocrine markers CD56 (-), SYN (diffuse +), CgA (-); other markers TTF-1 (diffuse +), NapsinA (-), P40 (diffuse +), NUT (nuclear punctate weak +), Ki67 (+60%). The FISH test result returned: 45% of tumor cells showed separation of NUT red and green signals (**Figure 2D**). The final pathological diagnosis: (right lung biopsy) poorly differentiated cancer, in line with NUT midline cancer. Genetic testing did not detect clinically significant types of genetic mutations. Whole exome sequencing (data raw data totaled 24.53G, average sequencing depth 89X) found a drug-related fusion gene neurotrophic receptor tyrosine kinase 2 (NTRK2). The whole-exome sequencing did not find the unique BRD4-NUT fusion gene of midline cancer.

Due to the large pulmonary lumps and obvious symptoms of chest pain at the time of treatment, our hospital was temporarily administered EP (etoposide + carboplatin) regimen for small cell lung cancer before the results of the pathological consultation in our hospital. After 1 cycle of chemotherapy, the patient had no chest tightness, and chest pain symptoms got better. After consulting the literature and the tumor MDT consultation in our hospital, the Ewing’s sarcoma SSG IX regimen was tried, and the VAI regimen (vincristine + epirubicin + ifosfone amide) and the PAI regimen (cisplatin + table) were applied respectively. Ruubicin + ifosfamide each chemotherapy cycle for 1 cycle, after the completion of 2 cycles of chemotherapy, the chest CT was reexamined to assess the reduction of tumor lesions (**Figure 1B**). After continuing to use the VAI regimen for 1 cycle of chemotherapy, the patient developed superior vena cava compression syndrome, edema of the face and upper extremities, difficulty breathing, and unable to lie supine (**Figure 1C**). Therefore, emergency radiotherapy was started on October 4, 2018, 1.5Gy/26 times, twice a day. The radiotherapy sites were right lung lesions, upper mediastinum, and right supraclavicular fossa lymph nodes. On October 11, 2018, the patient’s right lung tumor continued to progress (**Figure 1D**), and the radiotherapy ended, and a total of 13 radiotherapy sessions were performed. Subsequent treatment with Anlotinib (due to an allergic reaction and a widespread red rash on the upper body, the drug was discontinued), bevacizumab, paclitaxel, PD-1 inhibitor Corrida (Drug K), none of which could effectively control the disease progression (**Table 1**). The patient died on November 17, 2018, with an overall survival period of 4.7 months.

**Table 1:** The treatment received by the patient and its effect.

Time	Treatment programs	Curative effect
2018.07.10	Etoposide combined with carboplatin chemotherapy for 1 cycle	The patient’s chest tightness and chest pain have not improved
2018.08.01	SSG IV (VAI+PAI) VAI regimen (vincristine 2mg iv d1+ epirubicin 60mg iv d1-2+ ifosfamide 2g ivgtt d1-5) chemotherapy for 1 cycle; sequential PAI regimen (cisplatin 40mg d1-4+ epirubicin 60mgd1+Ifosfamide 2g ivgtt d1-5) 1 cycle of chemotherapy	The patient’s chest tightness and chest pain were significantly reduced compared with the previous, CT showed that the right lung mass was smaller than the previous, but the mediastinal lymph node was larger than the previous
2018.09.12	VAI regimen (vincristine 2mg iv d1+ epirubicin 60mg iv d1-2+ ifosfamide 2g ivgtt d1-5) chemotherapy for 1 cycle	The patient presented with superior vena cava compression syndrome, edema of the face and upper limbs, difficulty breathing, and unable to lie supine
2018.10.04	Start emergency radiotherapy, 1.5Gy/26 times, twice a day. The radiotherapy sites are right lung lesions, upper mediastinum, and right supraclavicular fossa lymph nodes	The patient’s local compression symptoms improved, and CT showed that the right lung mass was further enlarged
2018.10.13	Oral Anlotinib 12mg qd, stop taking the drug after 2 days due to allergic reaction	Notevaluated
2018.10.18	Bevacizumab 500mg intravenous infusion therapy	Not evaluated
2018.10.19	Paclitaxel 90mg intravenous chemotherapy	Not evaluated

## Discussion

NUT cancer is a rare malignant tumor that can occur at any age. The incidence is equal for men and women. It is more likely to occur in midline areas such as the larynx, nasal cavity, and mediastinum. It is also seen in organs such as the liver, pancreas, and bladder. It has a high degree of malignancy and a poor prognosis. Many patients have already metastasized tumors at the time of treatment, and the most common sites of metastasis are lymph nodes, bones, lungs, pleura, skin, and subcutaneous soft tissues (6, 9).

The histological origin of NUT cancer is unknown. Some studies (4, 10, 11) believe that the cell morphology of NUT cancer is more prone to squamous cell carcinoma, and there are reports that it is more prone to undifferentiated cancer. Hence, the pathological diagnosis of NUT cancer is particularly difficult. The histology of lung NUT carcinomas mostly presents the classic NUT carcinoma morphology, that is, the phenomenon of “sudden keratinization” appears in the background of undifferentiated small-medium-sized tumor cells (12). This case is a biopsy specimen. There is no sudden keratinization in morphology, and the cells are squeezed. Both SYN and TTF-1 are diffusely positively expressed, which makes it easy to misdiagnose as small cell carcinoma. At the same time, this type of tumor also expresses squamous cell carcinoma markers (P40 and CK5/6) and lung adenocarcinoma markers (TTF-1) (11, 13), and it is also easy to be misdiagnosed as lung squamous cell carcinoma and adenocarcinoma. NUT cancer is a highly aggressive tumor with rapid disease progression. It is extremely important to distinguish NUT cancer from other types of lung cancer. Moreover, NUT cancer is a rare tumor and is easily overlooked in clinical practice. Therefore, we have summarized and analyzed several pathological types in order to quickly identify NUT cancer. (1) Small cell carcinoma of the lung (14): The cell morphology is small and undifferentiated. Due to the small cell cytoplasm, it is prone to squeezing. These characteristics can overlap with NUT cancer, and the results of immunohistochemistry will also overlap. The difference between the two is mainly: small cell carcinoma expresses other neuroendocrine markers at the same time, such as CD56, CgA, NSE, etc. In most cases, it is diffusely strongly positive and does not express P40, P63, NUT and other markers. NUT cancer cells are mostly small to medium large and can express markers of squamous cell carcinoma, adenocarcinoma, and neuroendocrine cancer at the same time, and more than half of NUT cancers can express NUT. (2) Poorly differentiated squamous cell carcinoma (15): There is a certain overlap in morphology with NUT carcinoma, but most squamous cell carcinomas do not express TTF-1, SyN, and NUT. (3) Solid adenocarcinoma (16): Most cells have cytoplasm and do not express antibodies such as P40, SyN, and NUT. (4) Large cell carcinoma (4): The cells are too large, and the morphological and immunophenotypic characteristics can be diverse. This type of tumor is a diagnosis of exclusion and generally does not express P40, TTF-1, NUT, and other markers. (5) High-grade lymphoma (15): Sometimes, it is difficult to distinguish thisfrom NUT cancer by morphollogy, and it is easier to distinguish the two by immunohisto chemistry. For cases that are difficult to distinguish from NUT cancer in terms of morphology and immunohisto chemistry, FISH can be used to detect it.

Related literature reports that the median survival period of NUT cancer in various organs is 6 to 7 months, and the 1-year survival rate is 30% (4). However, case reports of lung NUT cancer (8, 17–22) suggest that lung NUT cancer is challengingto treat and has a poor prognosis (**Table 2**). The poor prognosis of NUT cancer is related to its rapid progression, easy recurrence, and unsatisfactory treatment effect. In most case reports of lung NUT cancer, even if the initial diagnosis is early and the operation is performed in time, the patient still relapses in a short time and progresses rapidly, and the OS is extremely short (13, 23). The prognosis of patients with multiple metastases throughout the body at the first diagnosis is worse (24). At the time of diagnosis, this patient had multiple lymph node metastases and lost the opportunity for surgery. So far, there is no standard treatment recommendation for NUT cancer (25). According to the literature report of Simone Storck’s sarcoma-based regimen for the treatment of children with NUT cancer (1), it was found that the use of SSG IV chemotherapy regimens to treat children with NUT cancer has achieved encouraging results. After failing to use the EP regimen, this patient was changed to SSG IV chemotherapy regimen. After 2 cycles of chemotherapy using VAI and PAI regimens, the patient’s chest tightness and chest pain were significantly improved, and the right lung mass was smaller than before, but the patient developed after 1 cycle superior vena cava compression syndrome. We found that although the SSG IV regimen has a certain therapeutic effect in the treatment of patients with NUT cancer, subsequent chemotherapy alone cannot maintain the therapeutic effect, leading to rapid disease progression. Giridharetal (26) retrospectively analyzed the prognostic relationship of 119 cases of NUT cancer and found that radiotherapy may benefit patients. In this case, after the occurrence of superior vena cava compression syndrome, local irradiation was performed on the right lung lesions, the upper mediastinum, and the right supraclavicular fossa lymph nodes at a dose of 1.5Gy/26 times, twice a day. Local compression symptoms were relieved, but the patient’s right lung lesions continued to increase, and radiotherapy was terminated. In this case, when the SSG IV regimen was used to treat the 2 cycles of lesion reduction, the combination of radiotherapy was not timely, and the subsequent radiotherapy could not control the progress of the disease. Therefore, the efficacy of SSG IV chemotherapy combined with radiotherapy in the treatment of NUT cancer is worthy of further study. The subsequent targeted therapy, immunotherapy, and chest cavity infusion chemotherapy failed to control tumor progression, and the overall survival time was 4.7 months, reflecting the highly aggressive nature of lung NUT cancer, similar to those reported in the literature.

**Table 2:** Summary of case reports of NUT cancer in recent years.

Case	Gender	Age(years)	Tumor Size(cm)	Treatment	OS(months)
1	M	48	7.7 × 7.4 × 6.0	Surgery	6
2	M	82	2.5 × 1.2 × 1.0	Surgery	NA
3	FM	21	5.7 × 4.5 × 5.4	Surgery and (pemetrexed+ lobaplatin)	5
4	M	39	12 × 6 × 7	(Carboplatin+nab-paclitaxel) and (doxorubicin + cyclophosphamide)and radiotherapy	NA
5	M	33	5.6× 5.1	(Pacli-taxelliposome+cisplatin) and radiotherapy and anlotinib	4
6	FM	63	NA	Unknown chemotherapy regimen	13+
7	M	23	5.5×4.1	Surgery +atezolizumab	1.5
8	M	53	5.4×3.7	(Paclitaxel-albumin+carboplatin)and Gefitinib and Apatinib	4.1
9	FM	30	4.7×4.7×4.7	(Paclitaxel-albumin+carboplatin)and paclitaxel-albumin and (paclitaxel-albumin+nivotuzumab)	3
10	M	25	10×6.4×12.7	Paclitaxel-albumin+carboplatin	1.5
11	M	74	NA	Radiotherapy and pembrolizumab	19.5
12	FM	58	NA	(Cetuximab+docetaxel+ platinum+radiotherapy)and (pembrolizumab+cetuximab) and (pembrolizumab+oxaliplatin)	26.7

Ex: F, Female; M, Male; NA, not available; 13m+, means surviving for more than 13 months.

In this case, genome-wide exome sequencing of this patient found the NTRK fusion gene. After consulting the CIViC database,Entrectinib and Larotrectinib NTRK for fusion geneare supported by the literature, and the recommended level is B. At the 2018 European Society of Medical Oncology (ESMO) conference, researchers reported the clinical data of NTRK’s targeted drug larotrectinib. In the preliminary clinical trials, for 109 patients using the targeted drug, the overall effective rate was 81%, and 17% of the patients had tumors disappeared completely (13).Larotrectinib was launched on November 27, 2018; however,this patient died on November 17 of the same year. The targeted drug was not used during the treatment of the patient, so its efficacy cannot be discussed. However, it is worth noting that in the future diagnosis and treatment of NUT cancer, we can do relevant genetic testing. If the patient carries the NTRK fusion gene, the corresponding targeted therapy can be tried.In addition, related research reports suggest that bromodomain and end motif (BET) inhibitors may be effective in treating NUT cancer. The results of phase I/II clinical trials of BET inhibitors (GSK525762 and NCT01587703) showed that 2 out of 10 NUT cancer patients had partial reactions, and 4 patients were in stable condition. In another clinical trial of the BET inhibitor OTX015/MK-8628, 3 out of 4 patients with NUT cancer had partial reactions (25, 27). Therefore, BET inhibitors may become a breakthrough drug in the treatment of NUT cancer in the future.

## Conclusion

In summary, reporting of this case helps us to further understand the pathological characteristics, treatment plan and research progress of NUT cancer. This case demonstrates that the SSG IV chemotherapy regimen has a certain effect on patients with NUT cancer,but chemotherapy alone cannot maintain the effect, and radiotherapy should be combined in the early stage. Targeted therapy and immunotherapy are not very effective for patients with NUT cancer. At present, the in-depth research of targeted therapy and the launch of NTRK targeted drugsmay bring better treatment options to patients. However, in future research, it is still necessary to further explore the pathogenesis and molecular genetic mechanism of the disease to improve the treatment and prognosis of patients.

## Data Availability Statement

The original contributions presented in the study are included in the article/supplementary material. Further inquiries can be directed to the corresponding author.

## Ethics Statement

Written informed consent was not obtained from the individual(s) for the publication of any potentially identifiable images or data included in this article.

## Author Contributions

RZ and ZH drafted and edited the manuscript. JW was the physician in charge of the patient and supervised the writing of the paper. XH, QZ and JZ co-supervised the writing of the paper. All the authors approved the final manuscript.

## Conflict of Interest

The authors declare that the research was conducted in the absence of any commercial or financial relationships that could be construed as a potential conflict of interest.

## Publisher’s Note

All claims expressed in this article are solely those of the authors and do not necessarily represent those of their affiliated organizations, or those of the publisher, the editors and the reviewers. Any product that may be evaluated in this article, or claim that may be made by its manufacturer, is not guaranteed or endorsed by the publisher.
